# Brain‐Wide Cellular‐Resolution Quantification of Amyloid Plaques and Microglia in AD Model Mice

**DOI:** 10.1002/alz.095690

**Published:** 2025-01-09

**Authors:** Damian G. Wheeler, Nathaniel Guanzon, Alexandria Murry, Emily May, Yessenia Gallegos, Chase Redd, Ricardo Azevedo, Sunil Gandhi

**Affiliations:** ^1^ Activity Signaling, LLC, San Diego, CA USA; ^2^ Translucence Biosystems, Inc, Irvine, CA USA; ^3^ Center for the Neurobiology of Learning and Memory, Department of Neurobiology and Behavior, University of California, Irvine, Irvine, CA USA

## Abstract

**Background:**

Recent advances in optical clearing and light sheet imaging have opened an exciting new avenue for brain‐wide, cellular resolution immunostaining at the forefront of a dimensional shift from 2D to 3D histology. When looking for read‐outs of genetic or pharmacological manipulations that affect the entire brain, traditional 2D immunohistochemistry approaches limit observations to brain regions of interest. Providing access to the intricate anatomy of the whole intact brain, tissue clearing offers neuroscientists unbiased and complete views of brain anatomy and function. One area where these methods have particular utility is in the development of CNS therapeutics where they can be used to examine the regional distribution of the therapeutics in the brain as well as brain‐wide target engagement and phenotypic efficacy. Toward this end, we have focused on Alzheimer’s Disease models developing clearing, imaging and quantification methods for brain‐wide regional quantification of amyloid plaques and microglia.

**Methods:**

Using our optimized iDISCO‐based tissue clearing method and our Mesoscale Imaging System for ZEISS Lightsheet microscopes, we can image micron‐scale resolution immunoreactivity across entire intact mouse brains in <20 min. Further, our AI‐powered Voxels software identifies individual immunostained cells and objects throughout the brain and aligns them to the Allen Reference Atlas to produce an unbiased, regionalized read‐out of cellular patterns across 100’s brain areas.

**Results:**

Using antibodies targeting the microglial protein Iba1, we can label microglia across the entire brain. We have developed multiple workflows to count microglia, provide metrics of shape and measure microglial activation. We have also trained machine learning algorithms to identify 6E10 antibody‐labeled β‐Amyloid throughout the brain and differentially quantify amyloid immunoreactivity in plaques and neuronal cell bodies. In 5xFAD Tg mice, rather than displaying the ramified morphology seen in WT mice, microglia become condensed and colocalize with β‐Amyloid plaques. Our automated methods segment and quantify these plaque‐associated microglia (PAMs) throughout the brain.

**Conclusion:**

These new methods for whole‐brain, next generation 3D immunohistochemistry are ideally suited to pre‐clinical studies for unbiased, complete and anatomically precise mapping of the efficacy of CNS therapeutics affecting amyloid deposition and neuroinflammation.

We thank NIMH for Grant R43MH122070.

